# Analysis of TET2 and EZH2 gene functions in chromosome instability in acute myeloid leukemia

**DOI:** 10.1038/s41598-020-59365-w

**Published:** 2020-02-17

**Authors:** Jingyi Wang, Na He, Ruiqing Wang, Tian Tian, Fengjiao Han, Chaoqin Zhong, Chen Zhang, Mingqiang Hua, Chunyan Ji, Daoxin Ma

**Affiliations:** 1 Department of Hematology, Qilu Hospital of Shandong University, Jinan, Shandong 250012 P.R. China; 2 Department of Hematology, Affiliated Hospital of Shandong University of Traditional Chinese Medicine, Jinan, 250011 P.R. China; 3 Department of Pathology and Microbiology, University of Nebraska Medical Center, Omaha, NE USA

**Keywords:** Cancer genetics, Acute myeloid leukaemia

## Abstract

TET2 and EZH2 play important roles in the epigenetic regulation in many cancers. However, their specific roles in acute myeloid leukemia (AML) pathogenesis remain unknown. Here, the expression, methylation or mutation of EZH2 and TET2 was determined and further correlated with the levels of the chromosome instability (CIN) genes MAD2 and CDC20. We down-regulated EZH2 and TET2 in AML cell lines and assessed the effect on CIN using fluorescence *in situ* hybridization (FISH). Our results showed that TET2, EZH2, MAD2 and CDC20 were aberrantly expressed in AML patients. The expression level of MAD2 or CDC20 was positively correlated with that of TET2 or EZH2. Hypermethylation of the TET2 gene down-regulated its transcription. Down-regulation of EZH2 or TET2 expression inhibited apoptosis, affected MAD2 and CDC20 expression, and promoted CIN in AML cells. Decitabine treatment restored TET2 methylation and EZH2 transcription and ameliorated CIN in AML. Therefore, TET2 and EZH2 play a tumor-inhibiting role in AML that affects CIN via MAD2 and CDC20.

## Introduction

AML comprises a heterogeneous group of malignancies in which immature and dysfunctional hematopoietic progenitors proliferate and accumulate in the bone marrow. Epigenetic modifications, including DNA methylation and histone methylation, have been shown to play a key role in the pathophysiology of AML^1–3^. Methylation at carbon atom 5 of the nucleotide cytosine (5-methylcytosine or 5-mC) usually induces inhibition of gene expression. Ten-eleven translocation methylcytosine dioxygenase 2 (TET2) is an evolutionarily conserved dioxygenase that can regulate gene transcription through DNA demethylation by catalyzing the conversion of 5-mC to 5-hydroxymethyl-cytosine (5-hmC)^4,5^. TET2 mutations are frequently acquired during the progression of myeloproliferative neoplasms (MPN) or myelodysplastic syndromes (MDS) to AML^6^. It was reported that TET2 down-regulation, independent of its mutational status, was correlated with poor survival in MDS or AML patients^7^. Moreover, a lower level of TET2 methylation can define a subgroup of AML that is highly curable and cannot be identified solely by genetic and cytogenetic analyses^8^. However, few studies regarding the correlation between the expression and the methylation status of TET2 in AML are reported.

Enhancer of zeste homolog 2 (EZH2), another epigenetic factor, is the catalytic component of polycomb repressive complex 2 (PRC2). EZH2 plays a controversial role in cancer development, acting as either an oncogene or a tumor suppressor depending on the type of cancer. Frequent mutations in the region encoding the catalytic SET domain of EZH2, such as the EZH2 (Tyr646) somatic mutation in humans or EZH2 (Tyr641) somatic mutation in mice, have been reported^9^. Somatic EZH2 mutations or amplifications are observed in many cancers, including prostate cancer, colon carcinoma and non-Hodgkin lymphoma. Gain-of-function somatic mutations of EZH2 (Tyr641) are frequently found in patients with follicular lymphoma and germinal center diffuse large B-cell lymphoma^10^. Overexpression of EZH2 has also been shown to play a critical role in various B cell tumors^11^. On the other hand, EZH2 inactivation in myeloid malignancies such as MDS or MDS/MPN is associated with poor prognosis and contributes to disease pathogenesis^12^. A recent study also showed that loss of EZH2 function induced multidrug resistance in AML^13^. However, the specific role of EZH2 in AML remains unclear.

The epigenetic factors TET2 and EZH2 can catalyze histone modification to alter chromatin structure and modulate gene expression. Therefore, deregulation of histone modification and acquisition of chromosome instability (CIN) due to abnormal expression, methylation or mutation of TET2 and EZH2 are crucial for AML development. CIN refers to the inability of cells to maintain correct chromosome complements after mitosis, resulting in aneuploidy. CIN usually arises at the mitotic checkpoint, and a key mediator of this pathway is the mitotic checkpoint protein MAD2. The expression level of MAD2 has been found to be significantly altered in many cancers^13^. Either elevated or reduced levels of MAD2 can cause abnormal mitotic checkpoint function in mice, resulting in CIN and tumor progression. Moreover, cell division cycle protein 20 homologue (CDC20), another mitotic checkpoint protein, is very important for chromosome segregation and spindle assembly. CDC20 regulates the anaphase-promoting complex or cyclosome (APC/C) ubiquitinase activity on its substrates and subsequent degradation of these proteins by proteasomes^14^. CIN promotes tumor development primarily through the aberrant expression of the mitotic checkpoint proteins MAD2 and CDC20. However, the specific roles of the CIN genes MAD2 and CDC20 in the pathogenesis of AML have not yet been identified.

To investigate the roles of the epigenetic/CIN/mitosis axis in AML, we determined the expression, methylation or mutation of TET2, EZH2, MAD2 and CDC20 and explored their clinical relevance in AML patients. Moreover, we down-regulated the epigenetic factors TET2 and EZH2 to further explore their therapeutic potential in AML.

## Materials and Methods

### Subjects and ethics statement

A total of 115 subjects were recruited in this study, comprising 58 AML patients who were newly diagnosed (ND) and 57 patients who were complete remission (CR), at Qilu Hospital of Shandong University (Jinan, China). The individual characteristics of these AML patients are shown in Supplementary Table 1. Because bone marrow aspiration is invasive, individuals with mild iron deficiency anemia were used as controls, which consisted of 15 subjects (age range, 18–67 years; median age, 39 years). This study was approved by the Medical Ethics Committee of Qilu Hospital of Shandong University. Written informed consent was obtained from each participant. Bone marrow mononuclear cells were isolated by the density-gradient centrifugation method. The detailed clinical characteristics of these individuals are summarized in Table 1.

**Table 1:** Clinical characteristics of AML patients and controls.

Clinical characteristics	ND AML patients (n = 58)	CR AML patients (n = 57)	Controls (n = 15)
Age (years, median, range)	43 (18–83)	40 (18–66)	39 (18–67)
Sex (male/female)	25/33	31/26	5/10
WBC (×10^9^/L)	42.51 ± 50.67	5.00 ± 2.40	5.50 ± 3.40
RBC (×10^12^/L)	2.44 ± 0.84	3.88 ± 0.86	3.93 ± 0.76
HGB (g/L)	78.79 ± 23.57	120.12 ± 26.44	121.32 ± 20.34
PLT (×10^9^/L)	60.98 ± 78.48	245.25 ± 124.15	255.25 ± 144.65
BM leukemic blasts (%)	79.81 ± 19.42	1.76 ± 0.68	
Abnormal karyotype (n, %)	16 (42%)	15 (75%)	
FAB classification			
M1	2	0	
M2	2	7	
M3	13	40	
M4	7	3	
M5	28	7	
unclassified	6	0	

Remarks: ND, newly diagnosed; CR, complete remission; WBC, white blood cells; RBC, red blood cells; HGB, haemoglobin; PLT, blood platelet; BM, bone marrow.

### Quantitative real-time polymerase chain reaction

Total RNA was abstracted from all samples using TRIzol reagent (Invitrogen, USA) according to the producer’s protocol. Then, cDNA was synthesized from approximately 1 µg of total RNA using a PrimeScript RT Reagent Kit (Perfect Real-Time (Takara Bio Inc., Japan). Reverse transcription was operated at 37 °C for 15 min followed by 85 °C for 5 s. Quantitative real-time PCR (qPCR) was performed using a LightCycler 480 II Real-time PCR system (Roche, Switzerland). The PCR mixture contained 5 μL of 2× SYBR Green Real-time PCR Master Mix, 1 μL of cDNA, 0.8 μL each of the forward and reverse primers, and 3.2 μL of ddH_2_O to make a final volume of 10 μL. The PCR primer sequences for the relevant genes are shown in Table 2. The results were expressed relative to the number of internal control GAPDH transcripts. Fold changes in mRNA expression levels were calculated using the comparative cycle threshold (Ct) method^15^.

**Table 2:** PCR primer sequences.

Gene	Forward primer (5′-3′)	Reverse primer (5′-3′)
EZH2	AATGTGGAGTGGAGTGGTGC	CGGGAGCTGGAGCTATGATG
MAD2	AGCTCCTTTTGACCTTCATTTC	TCCATTGCTTCATAGGTTCAAG
TET2	CCCACAGAGACTTGCACAACAT	CTGGCTCTGCTAACATCCTGAC
CDC20	GACATTCACCCAGCATCAAG	ATCCACGGCACTCAGACAG
GAPDH	GCACCGTCAAGGCTGAGAAC	TGGTGAAGACGCCAGTGGA
TET2-M	GCGTACGTGAATTTAAGGGTAC	AACAAAAAATCTCCACTACTACGAC
TET2-U	GGTGTATGTGAATTTAAGGGTATGT	AACAAAAAATCTCCACTACTACAAC
EZH2-ex16	TCATTGCAGAGGACCAACAC	TTCAGCCTGGACTTCTGCAT

### DNA methylation analysis of the TET2 promoter

Genomic DNA was isolated using a DNA Extraction Kit (Tiangen Biotech, Beijing, China) and was then chemically modified using an EZ DNA Methylation-Gold Kit (Zymo Research, Orange, CA, USA) according to the manufacturer’s instructions. The methylation status of the TET2 promoter was determined by methylation-specific PCR (MSP-PCR). As shown in Table 2, primers were designed to target unmethylated (U) and methylated (M) alleles to amplify the respective sequences using PCR. The PCR products were analyzed via agarose gel electrophoresis to determine the methylation status of TET2 based on the product size^16^.

### EZH2 mutation profile

Genomic DNA isolated from AML samples was used to amplify the mutated hotspots in EZH2 (E641 and Y646 on exon 16). The PCR mixture contained 12 µL of ddH_2_O, 1 µL of purified PCR product (10 ng/µL), 4 µL of BigDye (2.5×), 2 µL of BigDye Seq Buffer (5×) and 1 µL each of the forward and reverse primers to make up a final volume of 20 μL. The primer sequences are shown in Table 2. The cycling parameters consisted of an initial denaturation step at 94 °C for 2 min; 40 cycles of denaturation at 94 °C for 15 s, annealing at 56 °C for 30 s, and extension at 72 °C for 45 s; and a final extension step at 72 °C for 1 min. The PCR products were purified and subjected to bidirectional Sanger sequencing, and mutations were identified after comparison with the standard sequence^17^.

### Demethylase treatment with decitabine in AML cells

The AML cell lines HL-60, THP1 and U937 were cultured in RPMI 1640 medium (Gibco, USA) with 10% fetal bovine serum. The cells were incubated at 37 °C in humidified air containing 5% CO_2_. Demethylation was performed using decitabine (Xian Janssen, China). AML cells were seeded at a density of 1 × 10^6^ cells/mL in 6-well culture plates and treated with decitabine for 48 h. Then, cells were harvested for subsequent analysis.

### TET2 or EZH2 knockdown with siRNAs

TET2-siRNA, EZH2-siRNA and the respective controls were purchased from GenePharma (Shanghai, China). The siRNA was transfected into AML cells with Lipofectamine 2000 reagent (Invitrogen, Carlsbad, CA, USA) or Micropoly transfecter TM cell reagent (Micropoly, China) according to the manufacturer’s recommendation. The inhibition efficiency of the targeted genes was evaluated using qRT-PCR 24 h after transfection. The siRNA which has the highest knockdown efficiency was used for subsequent functional studies.

### Apoptosis assay

Apoptosis was detected using an Annexin V/fluorescein isothiocyanate (FITC) and propidium iodide (PI) apoptosis detection kit (BestBio, Shanghai, China). Briefly, after treatment with decitabine or transfection with TET2-siRNA or EZH2-siRNA for 48 h, 2 × 10^5^ cells were harvested, resuspended in 100 µl of flow cytometry binding buffer, and stained with 5 µL of Annexin V/FITC and 1 µL of PI. Then, the cells were incubated in the dark for 15 min at 4 °C, and 400 µL of binding buffer was added. The cells were immediately analyzed on a Galios flow cytometer (Beckman Coulter, CA, USA)^18^.

### CIN detection using fluorescence *in situ* hybridization (FISH)

Hybridization and FISH analysis were performed using standard procedures according to the published recommendations^18^. In brief, cells were hypotonically treated, fixed with methanol and acetic acid, and placed dropwise on slides. The fixed cells on the slides were hybridized with fluorescent probes against 5q, 7q, 20q12, +8, −Y, RB1, 1q21, D13S319 and IgH for 16 h. After being stained with DAPI, the cells were observed under a fluorescence microscope for measurement of the fluorescence signals. A total of 400 cells were counted for each sample, and the results were shown as the ratio of positive-signal cells to all counted cells.

### Statistical analysis

Data are presented as the means ± SDs or the medians (ranges). The statistical significance of differences between two groups was determined using two-sided t tests when the data were normally distributed or using the Mann-Whitney test when the data were non-normally distributed. Statistical analyses were performed using SPSS version 17.0 (SPSS, Chicago, IL, USA). Correlations between the epigenetic genes (TET2 or EZH2) and mitotic genes (MAD2 or CDC20) were analyzed using the Spearman correlation test. The value of the correlation coefficient indicates the presence or absence of correlation. P value less than 0.05 indicated statistical significance.

### Ethical approval

All procedures performed in studies involving human participants were in accordance with the ethical standards of the Medical Ethics Committee of Qilu Hospital of Shandong University and with the 1964 Helsinki declaration and its later amendments or comparable ethical standards.

### Informed consent

Informed consent was obtained from all individual participants included in the study.

## Results

### Epigenetic genes (TET2 and EZH2) and mitotic genes (MAD2 and CDC20) were aberrantly expressed in AML patients, and their levels restored after remission

**Figure 1:**
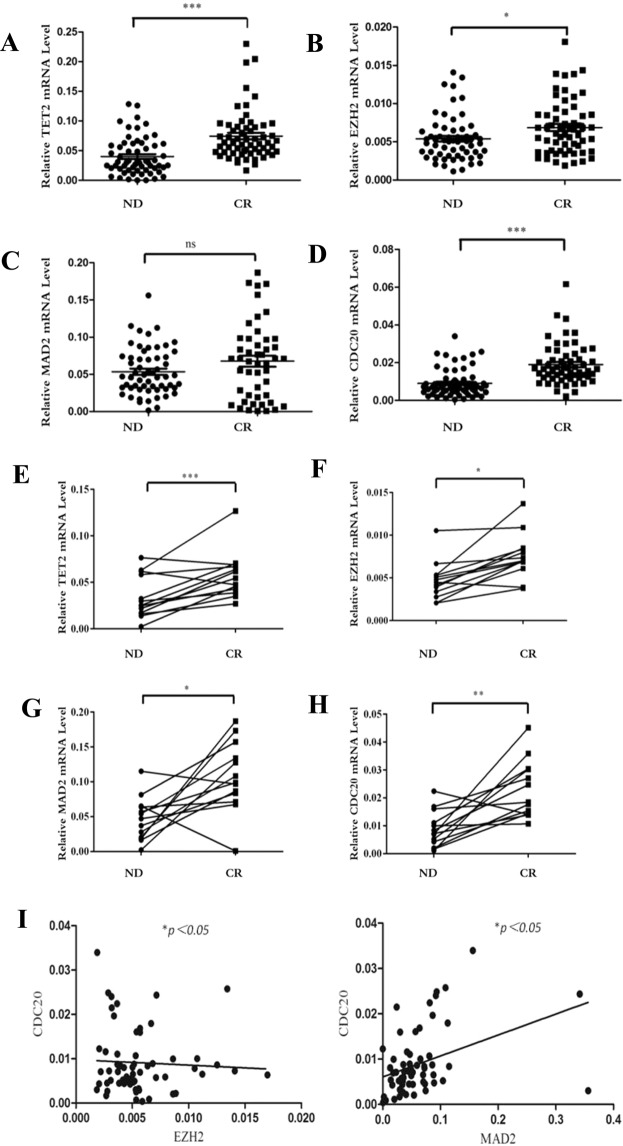
The mRNA expression results of EZH2, TET2, MAD2 and CDC20 in AML patients. (**A**–**D**) mRNA levels of EZH2, TET2, MAD2 and CDC20 in the whole ND/CR AML patients; (**E**–**H**), mRNA levels of EZH2, TET2, MAD2 and CDC20 in paired ND/CR AML patients; (**I**), The correlations between the levels of CDC20 and EZH2, CDC20 and MAD2. ***P < 0.0001, **P < 0.001, *P < 0.05, ns, no significance.

To investigate whether the epigenetic/CIN/mitosis axis participates in AML, we analyzed the mRNA expression of the related genes in AML patients using qRT-PCR. Compared to CR patients, ND AML patients had significantly down-regulated expression of TET2 (0.07456, 0.01686–0.2301 vs 0.04, 0.00004–0.1285; *P* < 0.0001), EZH2 (0.00686, 0.00191–0.01807 vs 0.0054, 0.00112–0.01408; *P* = 0.016) and CDC20 (0.01897, 0.00211–0.06164 vs 0.00898, 0.00038–0.03396, *P* < 0.0001), and marginally significantly decrease of MAD2 (0.06772, 0.00112–0.1869 vs 0.05359, 0.0016–0.156; *P* = 0.086) (Fig. 1A–D). In addition, to determine the changes in the expression of those genes during AML treatment, the whole treatment process of 13 AML patients was observed (ND-1 to ND-13 and CR-1 to CR-13 in Supplementary Table 1). CR was attained after standard induction chemotherapy. We observed significantly increased expression of TET2 (0.0252, 0.014–0.076 vs 0.0544, 0.027–0.127; *P* < 0.0001), EZH2 (0.00487, 0.00135–0.0123 vs 0.00689, 0.00465–0.0142; *P* = 0.0055), MAD2 (0.047, 0.002–0.115 vs 0.0988, 0.001–0.187; *P* = 0.0011) and CDC20 (0.00724, 0.001–0.22 vs 0.0185, 0.11–0.45; *P* = 0.0003) at the CR stage compared with the ND stage (Fig. 1E–H).

Moreover, correlations between the relative expression levels of the epigenetic genes and the mitotic genes were investigated. In ND AML patients, we observed a significantly positive correlation between the levels of EZH2 and CDC20 (r = 0.3867, *P* = 0.0305) and of MAD2 and CDC20 (r = 0.38672, *P* = 0.0087) (Fig. 1I). However, no significant correlation was found between the levels of EZH2 and MAD2 (*P* = 0.429) or between the levels of TET2 and either MAD2 or CDC20 (*P* = 0.145 and P = 0.144, respectively).

### Transcriptional down-regulation of TET2 may be associated with its hypermethylation status in AML

**Figure 2:**
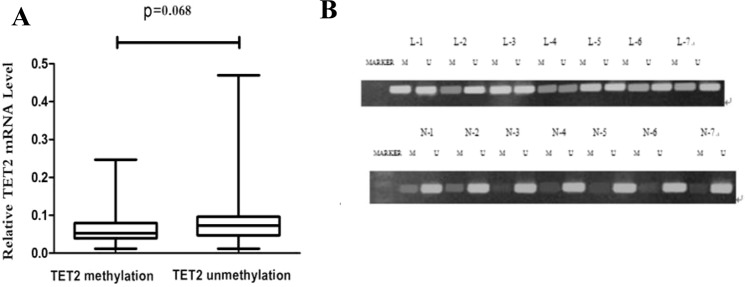
The methylation and expression levels of TET2 genes. (**A**) The expression level of TET2 in methylated AML patients was marginally lower than that in unmethylated ones. (**B**) Representative MSP results indicated the TET2 methylation status in AML samples and controls. TET2 was unmethylated in controls, but it was frequently hypermethylated in AML samples. M, methylated product; U, unmethylated product; N, control; L, acute myeloid leukemia.

To investigate whether promoter hypermethylation of TET2 resulted in reduced TET2 expression, we performed MSP to analyze TET2 methylation and qRT-PCR to measure TET2 expression in samples from 68 ND AML patients (Supplementary Table 2) and 15 controls. TET2 was hypermethylated in 21 of 68 (30.88%) AML samples, while no TET2 hypermethylation (0/15) was observed in the controls. The TET2 mRNA levels in hypermethylated AML samples displayed a marginal decrease compared with those in nonhypermethylated AML samples (0.053, 0.012–0.25 vs 0.087, 0.012–0.469; *P* = 0.068) (Fig. 2).

### EZH2 mutation profile

E641 and Y646 on exon 16 are mutation hotspots in EZH2. To investigate whether EZH2 is mutated in AML, we examined samples from 68 ND AML patients (Supplementary Table 2). However, neither of the mutated hotspots in exon 16 of EZH2 was found in our AML patients (Supplementary Fig. 1).

### Decitabine decreased TET2 methylation levels and induced the expression of TET2, EZH2, MAD2 and CDC20

**Figure 3:**
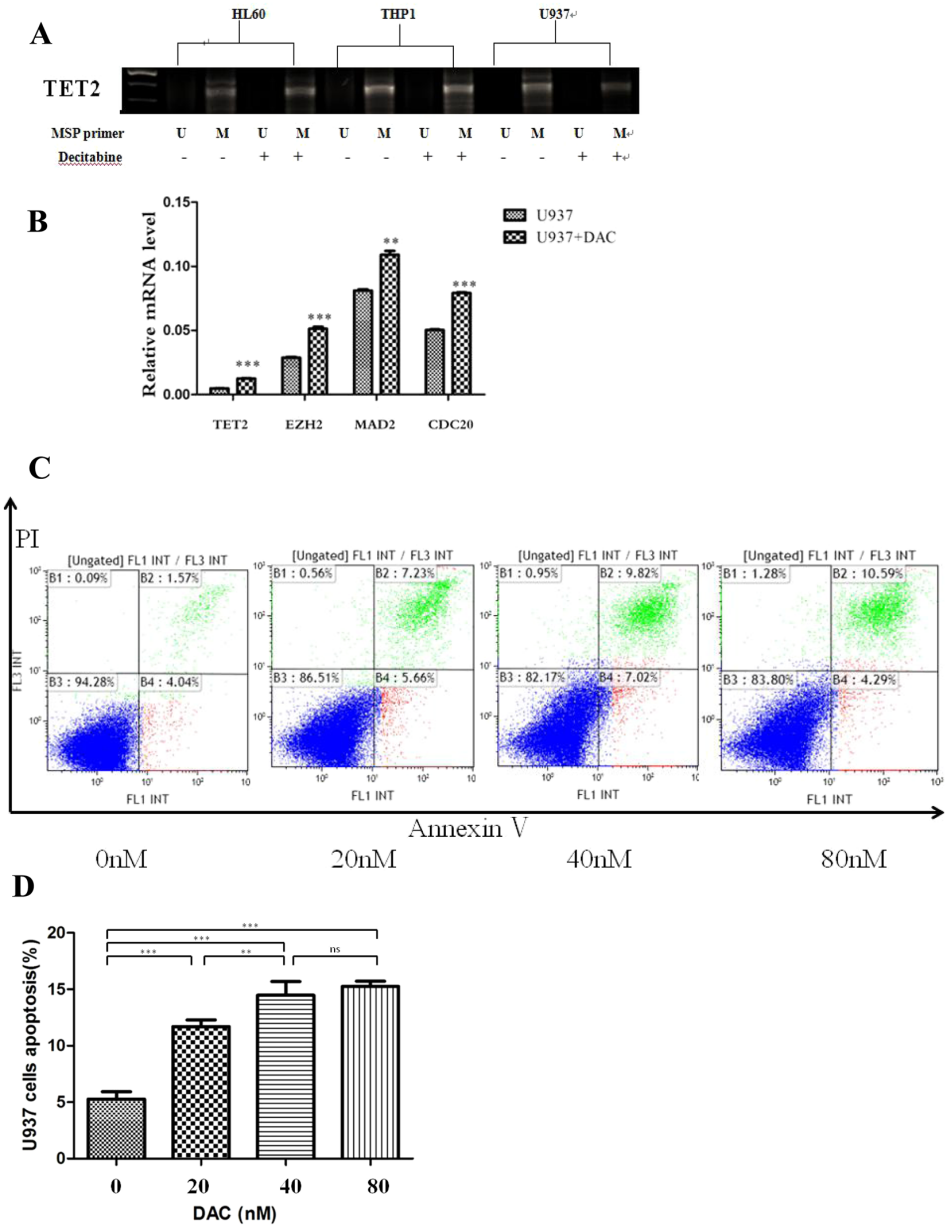
The effect of decitabine treatment. (**A**) The hypermethylation status of TET2 in HL60, THP1 and U937 cells after treatment of decitabine. M, methylated product; U, unmethylated product. (**B**) Decitabine treatment restored TET2, EZH2, MAD2 or CDC20 mRNA expression in U937 cells. (**C**,**D**) Decitabine treatment significantly increased the proportion of apoptotic cells relative to control treatment.

To determine the effect of decitabine on the methylation status of AML cells, we treated the 3 cell lines (HL60, THP1 and U937) with decitabine, then performed the TET2 methylation analysis. Our data showed that the demethylation effect of decitabine is more apparent in U937 cells compared with HL60 and THP1 (Fig. 3A). We also demonstrated that decitabine treatment upregulated the expression of TET2 (0.00487, 0.0047–0.005 vs 0.01247, 0.012–0.0131; P < 0.0001), EZH2 (0.029, 0.0276–0.0298 vs 0.05, 0.0496–0.0541; P = 0.0001), MAD2 (0.082, 0.0791–0.0821 vs 0.109, 0.103–0.112; P = 0.0009) and CDC20 (0.0508, 0.049–0.051 vs 0.0791, 0.0782–0.0801; *P* < 0.0001) in U937 cells (Fig. 3B). These findings indicated that decitabine might regulate the expression of TET2, EZH2, MAD2 and CDC20 by altering the methylation status of TET2. Moreover, we observed increased apoptosis of U937 cells upon decitabine treatment (Fig. 3C,D).

### Inhibition of either TET2 or EZH2 promoted the proliferation and reduced the apoptosis of AML cells

**Figure 4:**
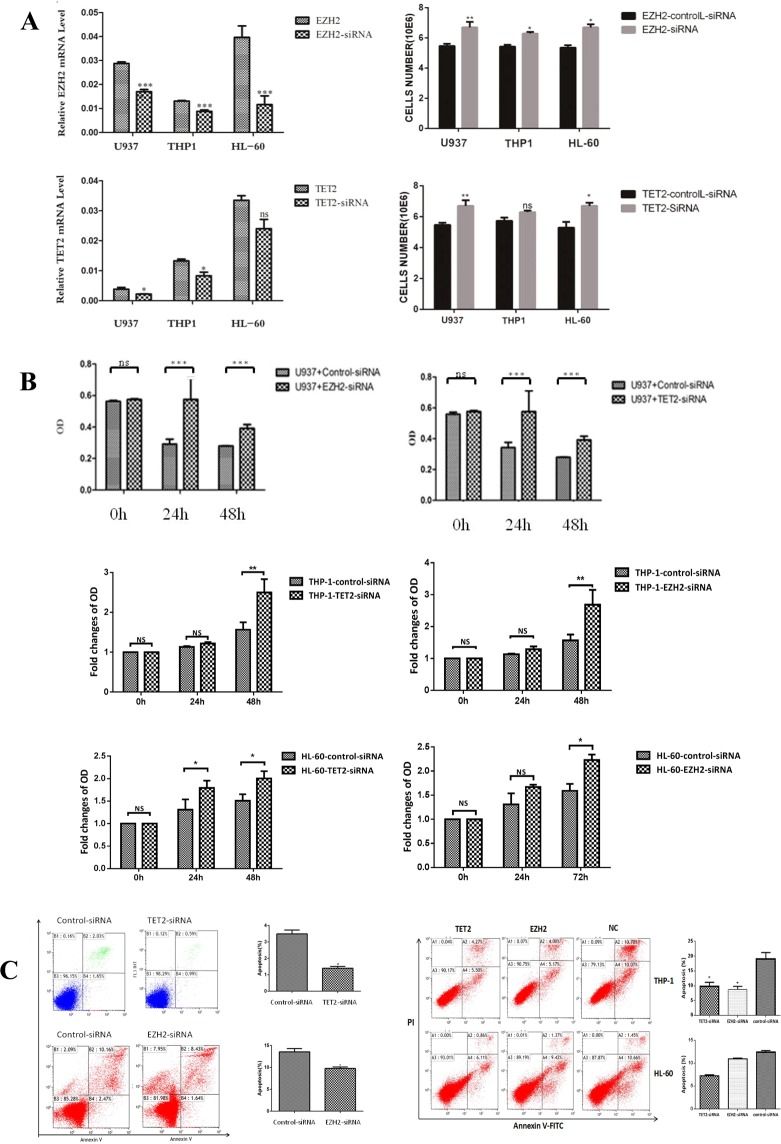
The results of EZH2/TET2 knockdown in AML cells. (**A**) The EZH2 and TET2 expressions and proliferation of U937, THP1 and HL-60 cells were assessed after siRNA transfection. (**B**) The proliferation results of AML cells by CCK8 method after EZH2/TET2 siRNA transfection. (**C**) The apoptosis results of EZH2/TET2-siRNA-transfected cells after siRNA transfection. *p < 0.05.

To evaluate the functional significance of epigenetic changes in AML, we knocked down TET2 and EZH2 via siRNA in AML cell lines (U937, THP1 and HL-60). Significant decreases in the EZH2 level in U937 (0.029, 0.0276–0.0298 vs 0.0165, 0.0157–0.0187; *P* < 0.001), THP1 (0.0131, 0.0128–0.0135 vs 0.0088, 0.0078–0.0094, *P* < 0.0001) and HL-60 (0.0396, 0.0337–0.049 vs 0.0116, 0.0062–0.0185, *P* < 0.0001) cells were observed after transfection (Fig. 4A). Similarly, the TET2 level in U937 (0.0043, 0.0027–0.0045 vs 0.0022, 0.002–0.0024; *P* < 0.05) and THP1 (0.0132, 0.0121–0.0141 vs 0.0083, 0.0062–0.0103, *P* < 0.05) was decreased significantly by siRNA. Our results obtained via the cell counting method that proliferation was inhibited in U937 cells more apparently than in THP1 or HL-60 cells (Fig. 4A). Then, we further demonstrated that the cell proliferation by CCK8 method was significantly increased (Fig. 4B) while apoptosis was significantly decreased in U937, THP1 and HL60 cells with TET2 or EZH2 down-regulation compared to control cells (Fig. 4C).

### Inhibition of EZH2 or TET2 can affect the expression of MAD2 and CDC20

**Figure 5:**
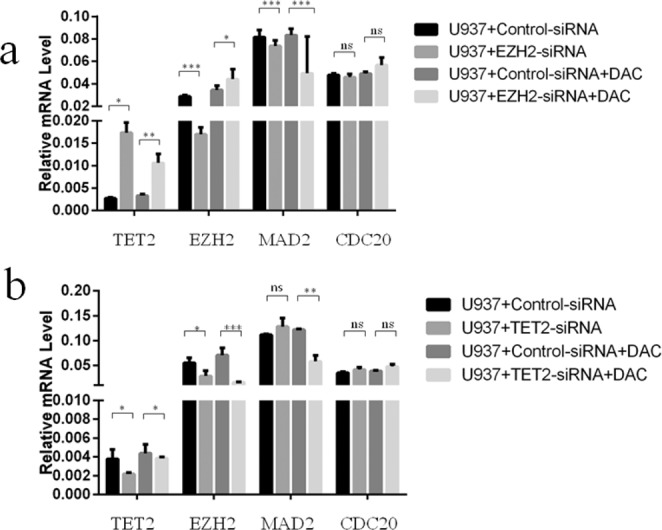
The gene expression results after EZH2 or TET2 down-regulation and decitabine treatment. (**a**) The gene expression results after EZH2 knock down and decitabine treatment. (**b**) The gene expression results after TET2 knock down and decitabine treatment.

We observed a significantly increased TET2 expression in EZH2-siRNA U937 cells compared to control cells (*P* < 0.05). Moreover, markedly decreased expression of MAD2 (0.0817, 0.0748–0.0872 vs 0.0738, 0.0693–0.0791, *P* < 0.0001) was found. However, no significant decrease in CDC20 (0.0479, 0.047–0.0494 vs 0.0459, 0.0436–0.049, *P* > 0.05) was found in EZH2-siRNA cells compared to control cells (Fig. 5a). Similarly, in TET2-siRNA cells, EZH2 expression was significantly decreased (*P* < 0.05). However, no significant increase was found in the expression level of MAD2 (0.1116, 0.1103–0.1134 vs 0.1278, 0.1127–0.1466, *P* > 0.05) or CDC20 (0.035, 0.0326–0.0364 vs 0.0415, 0.0382–0.0464, *P* > 0.05) in TET2-knockdown cells (Fig. 5b).

### Decitabine restored the siRNA-induced reduction in TET2 or EZH2 expression in U937 cells

After decitabine treatment, we observed a significant increase in the levels of TET2 (0.0023, 0.0022–0.0025 vs 0.0039, 0.0038–0.004; *P* < 0.0001) or EZH2 (0.0166, 0.0157–0.0187 vs 0.0487, 0.0342–0.0497; *P* = 0.0059) in TET2- or EZH2-siRNA U937 cells compared with decitabine-untreated control cells. However, decitabine reduced MAD2 expression in TET2-siRNA U937 cells and did not significantly affect other genes (Fig. 5).

### Inhibition of EZH2 or TET2 affects CIN in U937 cells

**Figure 6:**
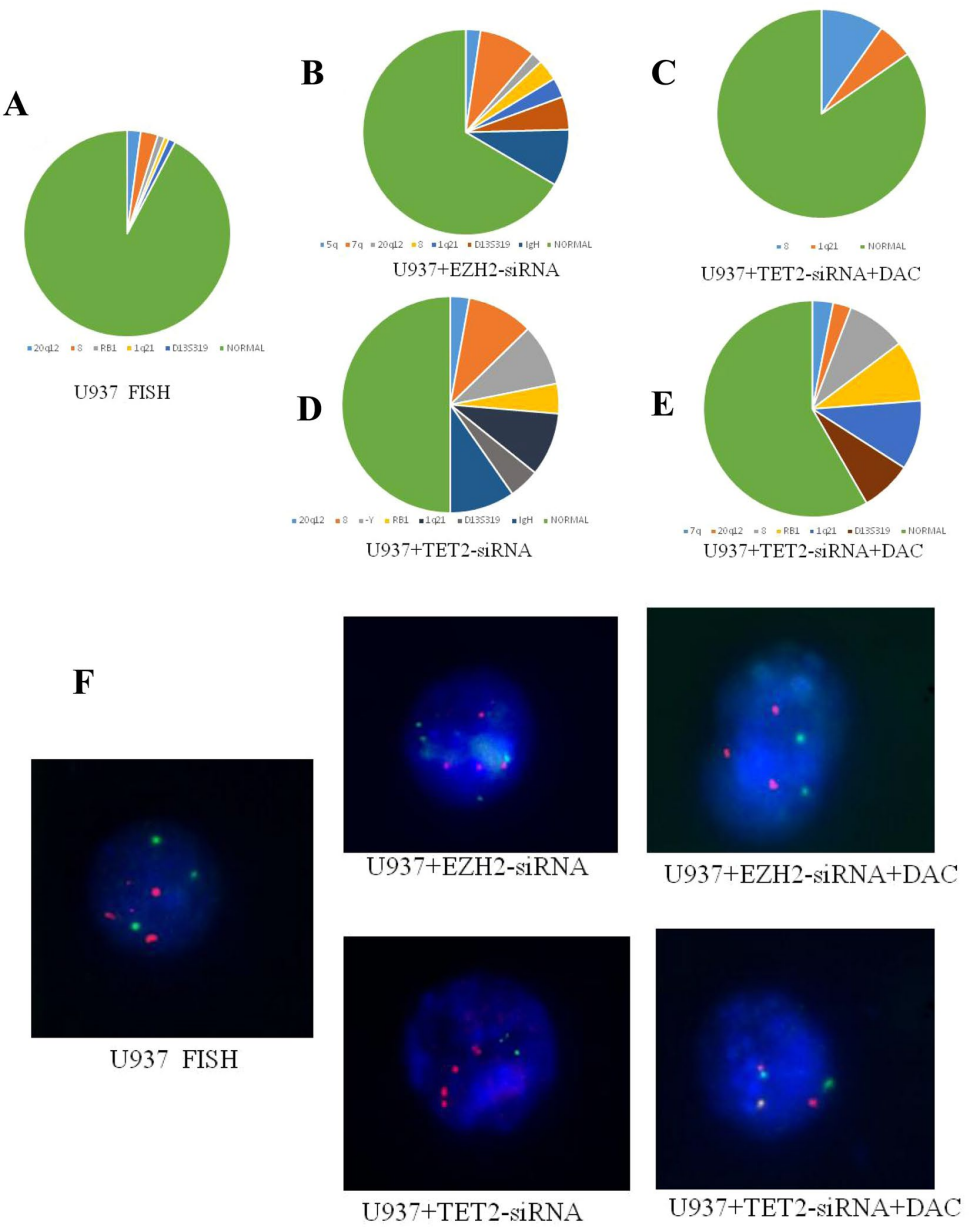
The FISH results after EZH2 or TET2 down-regulation or decitabine treatment. (**A**) There were 5 abnormally marked positions in U937 cells as follows: +8 (2.7%), 20q12 (2.1%), D13S319 (1.1%), RB1 (1.1%), and 1q21 (0.67%). (**B**) EZH2-siRNA-transfected U937 cells had 7 abnormally marked positions: 7q (8.9%), D13S319 (5.2%), +8 (3.3%), 1q21 (3.1%), 5q− (2.3%), 20q12− (1.8%), and IgH (8.9%). (**C**) After decitabine treatment of EZH2-siRNA-transfected U937 cells, the number of abnormally marked positions decreased: 1q21 (5.6%), and +8 (9.7%). (**D**) TET2-siRNA-transfected U937 cells had 7 abnormally marked positions: +8 (10.8%), IgH (10.6%), 1q21 (10.4%), D13S319 (5%), RB1 (4.9%), and 20q12 (3.1%). (**E**) After decitabine treatment, the number of abnormally marked positions in TET2-siRNA-transfected U937 cells decreased: 1q21 (10.3%), RB1 (9.1%), +8 (8.9%), D13S319 (7.6%), 7q (3.1%), and 20q12 (2.7%). (**F**) The typical representative pictures of FISH.

To explore the effect of EZH2 and TET2 on chromatin structure, we performed FISH in decitabine-treated or untreated U937 cells using probes against 5q−, 7q−, 20q12−, +8, –Y, RB1, 1q21, D13S319 and IgH, which were used as markers of CIN. We found five chromosomal abnormalities in U937 cells, which were increased to seven in EZH2-siRNA-transfected U937 cells and seven in TET2-siRNA-transfected U937 cells and were decreased after decitabine treatment (Fig. 6A–F). These data indicated that knockdown of EZH2 or TET2 induced an increase in the level of CIN compared with that in control cells and this increase can be reversed by decitabine treatment.

## Discussion

Epigenetic dysregulation caused by aberrant DNA methylation or histone modification plays an important role in the pathogenesis of AML. It is reported that epigenetic silencing of many novel potential tumor suppressor genes contributes to AML. Recently, TET2 has been considered as a novel tumor suppressor, and TET2 mutations have been demonstrated to be acquired in 12–24% of myeloid neoplasms, including AML, MDS, CMML and MPN^19^. Moreover, it has been shown that silencing TET2 via hypermethylation is a common pathogenetic mechanism in different cancers. The methylation status of TET2 promoter was also assessed in pediatric patients with ALL and was found to be positive in only one B-ALL patient^20^. This finding indicates that the methylation status of TET2 can affect the pathogenesis of acute leukemia.

However, there are few studies regarding the methylation status and functions of TET2 in AML. In our study, we observed hypermethylated TET2 in 21 of 68 (30.88%) AML patients but in none of the controls. Moreover, there was a marginal decrease in TET2 mRNA levels in AML patients with TET2 hypermethylation compared to those without TET2 hypermethylation. Our data showed that the hypermethylation status of TET2 may contribute to the decrease in TET2 expression in AML patients. Furthermore, there is rare study about the role of TET2 methylation in tumor prognosis. In our manuscript, because of the short-term follow-up of the AML patients, we have no survival data of these patients and cannot further analyze the correlation between methylation status and prognosis. If possible, we will follow up these patients and do this correlation analysis in the future.

Suppression of EZH2 expression was reported to induce chemoresistance in AML cell lines and primary cells. Decreased EZH2 levels can result in derepression of the HOX gene, and inhibiting expression of HOXB7 and HOXA9 in resistant cells could enhance cellular sensitivity to tyrosine kinase inhibitors and other chemotherapeutic drugs. These suggested that restoration of EZH2 was a potential method to overcome chemoresistance in AML^21^. However, it was also proved that EZH2 as an oncogenic mutation induced lymphoma and melanoma through a vast reorganization of chromatin structure^22^. EZH2 plays dual roles in malignant diseases. To investigate the specific role of EZH2 in AML, we examined samples from 68 newly diagnosed patients with AML. However, neither of the mutated hotspots in EZH2 exon 16 was found in any of these patients. A significant increase was found in the expression of EZH2 at CR stage compared with that at ND stage, suggesting the mitigation of dys-regulated gene expression after chemotherapy.

In the process of mitosis, the duplicated chromosomes are split and evenly scattered to progeny cells leading by the spindle. Many researches indicated a mitotic checkpoint which inhibits chromosome segregation until all of the chromosomes are appropriately affixed to microtubules by kinetochores. Many kinetochore-localized proteins, including MAD2 and CDC20, have been involved in this process. Abnormal expression of MAD2 and CDC20 was reported to induce tumor cell aneuploidy in various malignancies. However, MAD2 levels vary significantly in human tumors^23,24^, and studies in animals indicated that either high or low MAD2 levels could induce aberrant mitotic checkpoint functions, resulting in CIN and tumor progression^25,26^. MAD2 primarily senses the connection between microtubules and the centromere. During mitosis, MAD2 recruits CDC20 to form the MAD2-CDC20 complex, which accumulates and thus inhibits APC/C activity to ensure normal mitosis. Thus, either low or high expression of MAD2 or CDC20 can lead to aneuploidy and even tumor formation. In the present study, we observed a positive relationship between the EZH2 and MAD2/CDC20 levels. In U937 cells, interestingly, TET2 expression increased in response to EZH2 knockdown, while the EZH2 level decreased with knockdown of TET2. This interesting phenomenon may be due to the dual function of EZH2. On the other hand, we considered that TET2 may be upstream of EZH2 and that negative feedback from EZH2 expression leads to the increased TET2 mRNA expression. This observation indicates that the balance of TET2 and EZH2 mRNA expression is important for the proliferation and apoptosis of AML cells. Further research is needed to explore the mechanism.

Many ongoing studies focused on the CIN inhibitors. The role of CIN in hematological malignancies is currently an attractive topic. Though it is widely accepted that aberrant CIN promotes the proliferation and survival of AML cells, the interactions between epigenetic regulation and CIN are not fully understood in AML. Our results showed that knockdown of either EZH2 or TET2 induced an increase in the level of CIN compared with that in controls in U937 cells. Decitabine treatment of EZH2- or TET2-siRNA-transfected U937 cells decreased the number of abnormally marked positions. These results suggest that EZH2 and TET2 can act antagonistically to regulate cell growth by modulating CIN by influencing MAD2 or CDC20 expression in AML.

In summary, down-regulation of EZH2 or TET2 affected cell proliferation and influenced MAD2 or CDC20 expression, ultimately ameliorating CIN. Decitabine may partly ameliorate these abnormal changes. Further studies to clarify the specific roles of these mediators in AML are awaited and will ultimately provide a perspective for clinical treatment^16,17,27^.

## Supplementary information

Dataset 1.
