# Supplementary material for: Analysis of TET2 and EZH2 gene functions in chromosome instability in acute myeloid leukemia
Source: Sci Rep. 2020 Feb 17;10:2706. doi: 10.1038/s41598-020-59365-w (PMC7026035; doi:10.1038/s41598-020-59365-w)
Supplement: Supplementary file 1 — Dataset 1. [file 41598_2020_59365_MOESM1_ESM.docx]

Analysis of TET2 and EZH2 gene functions in chromosome instability in acute myeloid leukemia

Jingyi Wang^1,2#^; Na He^1#^, Ruiqing Wang^1#^,Tian Tian^3^, Fengjiao Han^1^, Chaoqin Zhong^1^, Chen Zhang^1^, Mingqiang Hua^1^, Chunyan Ji^1^, Daoxin Ma^1*^

1. Department of Hematology, Qilu Hospital of Shandong University, Jinan, Shandong 250012, P.R. China

2. Department of Hematology, Affiliated Hospital of Shandong University of Traditional Chinese Medicine, Jinan 250011, P.R. China

3. Department of Pathology and Microbiology, University of Nebraska Medical Center, Omaha, NE, U.S.A.

# These authors contributed equally to this work.

**Supplementary Table 1. The individual characteristics of AML patients for expression determination**

| Number | Name | Ages | Gender | Diagnose | WBC  10^9^/L | RBC  10^12^/L | HGB  g/L | PLT  10^9^/L | BLAST  % |
| --- | --- | --- | --- | --- | --- | --- | --- | --- | --- |
| ND-1 | WJR | 34 | F | M5 | 13 | 3.6 | 60 | 21 | 95 |
| ND-2 | XSM | 83 | F | M5 | 56.97 | 3.1 | 103 | 29 | 95 |
| ND-3 | LJF | 61 | F | M5 | 96.2 | 1.17 | 55 | 8 | 35 |
| ND-4 | ZZH | 22 | M | M4 | 36.16 | 1.49 | 54 | 21 | 93 |
| ND-5 | ZL | 40 | M | M5 | 45.23 | 2.01 | 77 | 61 | 80 |
| ND-6 | SSS | 38 | F | M3 | 2.15 | 4.31 | 142 | 128 | 79 |
| ND-7 | CXH | 28 | M | M5 | 14.59 | 3.19 | 96 | 21 | 81 |
| ND-8 | XYP | 53 | F | M3 | 17.46 | 2.79 | 89 | 10 | 90 |
| ND-9 | SY | 46 | M | M5 | 49.41 | 2.4 | 74 | 28 | 96 |
| ND-10 | GHY | 21 | F | M5 | 3.76 | 1.95 | 66 | 39 | 95 |
| ND-11 | LXJ | 53 | M | M4 | 65.87 | 1.14 | 57 | 110 | 63 |
| ND-12 | SQC | 52 | M | M3 | 31.14 | 2.34 | 73 | 51 | 85 |
| ND-13 | WJM | 34 | F | M3 | 190 | 2.74 | 102 | 54 | 95 |
| ND-14 | YYS | 51 | M | M5 | 9.56 | 1.25 | 46 | 30 | 78 |
| ND-15 | HYQ | 49 | F | M5 | 89.17 | 3.46 | 93 | 126 | 91 |
| ND-16 | LLM | 57 | M | M3 | 92.21 | 2.86 | 79 | 64 | 93 |
| ND-17 | ZYJ | 17 | F | M5 | 356 | 1.77 | 57 | 41 | 86 |
| ND-18 | MGQ | 25 | F | M3 | 12 | 3 | 80 | 60 | 94 |
| ND-19 | JK | 40 | M | M5 | 11 | 2 | 75 | 65 | 90 |
| ND-20 | CXF | 21 | F | AML | 14.09 | 4 | 66 | 77 | 80 |
| ND-21 | ZY | 43 | F | M1 | 45.59 | 2.63 | 89 | 48 | 42 |
| ND-22 | LWQ | 43 | M | M3 | 54.52 | 2.22 | 60 | 60 | 37 |
| ND-23 | LYG | 50 | F | M3 | 29.3 | 2.14 | 81 | 56 | 96 |
| ND-24 | LXJ | 41 | M | AML | 32.47 | 3.22 | 109 | 17 | 98 |
| ND-25 | ZYJ | 57 | F | M4 | 72.81 | 1.98 | 70 | 53 | 86 |
| ND-26 | LXL | 17 | F | M2 | 48.93 | 3.94 | 129 | 24 | 64 |
| ND-27 | YN | 29 | F | M3 | 6.09 | 4.4 | 135 | 191 | 34 |
| ND-28 | ZKG | 25 | M | M2 | 3.93 | 2.06 | 68 | 35 | 28 |
| ND-29 | LLH | 83 | M | AML | 6 | 3 | 67 | 78 | 78 |
| ND-30 | JWB | 43 | M | AML | 19.67 | 2.39 | 89 | 506 | 49 |
| ND-31 | KFY | 45 | M | M3 | 4.26 | 2.78 | 84 | 10 | 91 |
| ND-32 | WHR | 68 | F | M4 | 173.41 | 3.51 | 100 | 22 | 93 |
| ND-33 | ZL | 59 | F | AML | 1.5 | 2.2 | 65 | 72 | 43 |
| ND-34 | DW | 34 | F | M5 | 19.25 | 1.86 | 65 | 47 | 78 |
| ND-35 | QYH | 33 | M | M3 | 73.06 | 2.55 | 90 | 37 | 96 |
| ND-36 | ZHM | 57 | F | M4 | 223.4 | 2.77 | 82 | 35 | 82 |
| ND-37 | WF | 43 | F | M5 | 16.26 | 1.35 | 55 | 6 | 90 |
| ND-38 | LYX | 29 | F | M5 | 46.51 | 1.03 | 57 | 35 | 91 |
| ND-39 | LKL | 44 | F | M5 | 1.21 | 2.31 | 75 | 106 | 62 |
| ND-40 | LXY | 65 | F | M5 | 16.13 | 2.05 | 65 | 105 | 81 |
| ND-41 | LWJ | 46 | M | M3 | 104.89 | 2 | 66 | 78 | 93 |
| ND-42 | WYL | 13 | F | M3 | 1.63 | 2.34 | 75 | 33 | 92 |
| ND-43 | LY | 55 | M | M1 | 2.73 | 2.37 | 85 | 8 | 92 |
| ND-44 | WXY | 23 | F | M5 | 2.2 | 1.83 | 65 | 35 | 90 |
| ND-45 | XGX | 18 | F | M4 | 14.57 | 2.18 | 70 | 187 | 49 |
| ND-46 | LHL | 46 | F | M5 | 15.05 | 3.38 | 115 | 43 | 85 |
| ND-47 | JYX | 39 | M | M3 | 5.38 | 3.49 | 92 | 10 | 92 |
| ND-48 | ZY | 42 | F | M3 | 0.8 | 1.99 | 71 | 23 | 80 |
| ND-49 | PZQ | 43 | M | AML | 54.12 | 2.22 | 66 | 76 | 37 |
| ND-50 | WCJ | 44 | M | M5 | 1.21 | 2.01 | 80 | 90 | 62 |
| ND-51 | LJT | 65 | M | M5 | 17.13 | 2.05 | 65 | 115 | 80 |
| ND-52 | WXH | 45 | F | M5 | 7.57 | 2.23 | 87 | 44 | 90 |
| ND-53 | CL | 46 | F | M4 | 39.58 | 3.45 | 86 | 24 | 90 |
| ND-54 | GFS | 28 | M | M3 | 2.78 | 2.09 | 68 | 44 | 92 |
| ND-56 | RW | 37 | F | M5 | 32.28 | 1.61 | 59 | 59 | 95 |
| ND-57 | YCL | 32 | F | M3 | 5.3 | 1.42 | 65 | 8 | 88 |
| ND-58 | JZZ | 77 | F | M5 | 2.71 | 1.77 | 68 | 12 | 84 |
| CR-1 | WJR | 34 | F | M5 | 5.94 | 4.47 | 140 | 226 | 3 |
| CR-2 | XSM | 83 | F | M5 | 5.14 | 3.98 | 121 | 456 | 2 |
| CR-3 | LJF | 61 | F | M5 | 2.8 | 3.85 | 108 | 82 | 1 |
| CR-4 | ZZH | 22 | M | M4a | 5.29 | 1.79 | 63 | 272 | 3 |
| CR-5 | ZL | 40 | M | M5 | 4.99 | 4.1 | 119 | 260 | 2 |
| CR-6 | SSS | 38 | F | M3a | 2.99 | 3.38 | 98 | 142 | 3 |
| CR-7 | CXH | 28 | M | M5 | 5.54 | 5.23 | 161 | 201 | 4 |
| CR-8 | XYP | 53 | F | M3a | 6.15 | 4.49 | 115 | 368 | 1 |
| CR-9 | SY | 46 | M | M5 | 4.78 | 3.48 | 110 | 116 | 2 |
| CR-10 | GHY | 21 | F | M5 | 2.6 | 3.3 | 112 | 91 | 5 |
| CR-11 | LXJ | 53 | M | M4b | 4.8 | 4.4 | 139 | 229 | 3 |
| CR-12 | SQC | 52 | M | M3 | 4.8 | 4.4 | 139 | 229 | 3 |
| CR-13 | WJM | 34 | F | M3 | 4.15 | 2.66 | 84 | 597 | 4 |
| CR-14 | WJR | 34 | F | M3 | 5.07 | 4.3 | 132 | 254 | 5 |
| CR-15 | WXJ | 34 | F | M2b | 7.32 | 4.02 | 129 | 185 | 1 |
| CR-16 | MYN | 53 | M | M3 | 5.59 | 4.16 | 122 | 413 | 2 |
| CR-17 | YZY | 44 | M | M3 | 6.32 | 3.37 | 101 | 362 | 3 |
| CR-18 | SYM | 18 | M | M4 | 8.26 | 3.42 | 114 | 583 | 3 |
| CR-19 | CK | 56 | M | M3 | 5.94 | 4.47 | 140 | 226 | 3 |
| CR-20 | NZH | 27 | M | M3 | 5.14 | 3.98 | 121 | 456 | 2 |
| CR-21 | MDZ | 41 | M | M3 | 2.8 | 3.85 | 108 | 82 | 1 |
| CR-22 | MZR | 66 | F | M3 | 5.29 | 1.79 | 63 | 272 | 3 |
| CR-23 | LJF | 39 | M | M3 | 2.99 | 3.38 | 98 | 142 | 3 |
| CR-24 | LJ | 62 | M | M2b | 5.54 | 5.23 | 161 | 201 | 4 |
| CR-25 | WHX | 35 | M | M2 | 6.15 | 4.49 | 115 | 368 | 1 |
| CR-26 | LH | 64 | M | M3 | 4.78 | 3.48 | 110 | 116 | 2 |
| CR-27 | YKL | 43 | F | M3 | 2.6 | 3.3 | 112 | 91 | 5 |
| CR-28 | LXP | 26 | F | M3 | 4.8 | 4.4 | 139 | 229 | 3 |
| CR-29 | YN | 55 | F | M3 | 4.8 | 4.4 | 139 | 229 | 3 |
| CR-30 | CWQ | 26 | F | M3 | 4.15 | 2.66 | 84 | 597 | 4 |
| CR-31 | HJJ | 29 | F | M2 | 5.07 | 4.3 | 132 | 254 | 5 |
| CR-32 | WJN | 28 | M | M3 | 7.32 | 4.02 | 129 | 185 | 1 |
| CR-33 | MMM | 28 | M | M3 | 5.59 | 4.16 | 122 | 413 | 2 |
| CR-34 | ZR | 31 | F | M3 | 6.32 | 3.37 | 101 | 362 | 3 |
| CR-35 | MRF | 59 | F | M3 | 8.26 | 3.42 | 114 | 580 | 3 |
| CR-36 | NSE | 21 | F | M3 | 5.94 | 4.47 | 140 | 226 | 3 |
| CR-37 | ST | 34 | M | M2 | 5.14 | 3.98 | 121 | 326 | 2 |
| CR-38 | ZL | 19 | M | M3 | 2.8 | 3.85 | 108 | 82 | 1 |
| CR-39 | WQH | 47 | F | M2 | 5.29 | 1.79 | 120 | 252 | 3 |
| CR-40 | ZQS | 51 | F | M3 | 2.99 | 3.38 | 135 | 142 | 3 |
| CR-41 | ZCF | 18 | F | M3 | 5.54 | 5.23 | 161 | 201 | 4 |
| CR-42 | LWJ | 43 | M | M3 | 6.15 | 4.49 | 115 | 168 | 1 |
| CR-43 | JYX | 47 | M | M3 | 4.78 | 3.48 | 110 | 116 | 2 |
| CR-44 | ZXN | 41 | M | M3 | 2.6 | 3.3 | 112 | 91 | 5 |
| CR-45 | FQM | 47 | M | M3 | 5.94 | 4.47 | 140 | 226 | 3 |
| CR-46 | XLG | 22 | F | M3 | 5.14 | 3.98 | 121 | 200 | 2 |
| CR-47 | SMS | 25 | F | M3 | 2.8 | 3.85 | 108 | 182 | 1 |
| CR-48 | ZXN | 33 | M | M3 | 5.29 | 1.79 | 120 | 272 | 3 |
| CR-49 | ZGY | 30 | M | M3 | 2.99 | 3.38 | 98 | 140 | 3 |
| CR-50 | ZFK | 18 | M | M3 | 5.54 | 5.23 | 161 | 201 | 4 |
| CR-51 | ZYJ | 38 | M | M3 | 6.15 | 4.49 | 115 | 260 | 1 |
| CR-52 | ZZH | 29 | M | M3 | 4.78 | 4.48 | 110 | 111 | 2 |
| CR-53 | CM | 41 | F | M2b | 2.6 | 3.3 | 112 | 99 | 5 |
| CR-54 | LYG | 46 | M | M3 | 4.8 | 4.4 | 139 | 229 | 3 |
| CR-55 | LXY | 32 | F | M3 | 4.8 | 4.4 | 139 | 229 | 3 |
| CR-56 | RW | 51 | M | M3 | 5.54 | 5.23 | 161 | 201 | 4 |
| CR-57 | WJM | 58 | F | M3 | 6.15 | 4.49 | 115 | 268 | 1 |

Remarks: ND, newly diagnosed; CR, complete remission; WBC, white blood cells; RBC, red blood cells; HGB, haemoglobin; PLT, blood platelet; BM, bone marrow.

**Supplementary Table 2. The individual characteristics of ND AML patients for the determination of TET2 hypermethylation and EZH2 mutation**

| Number | NAME | Ages | Gender | Diagnose | WBC  10^9^/L | RBC  10^12^/L | HGB  g/L | PLT  10^9^/L | BLAST  % |
| --- | --- | --- | --- | --- | --- | --- | --- | --- | --- |
| ND-1 | WJR | 34 | F | M5 | 13 | 3.6 | 60 | 21 | 95 |
| ND-2 | XSM | 83 | F | M5 | 56.97 | 3.1 | 103 | 29 | 95 |
| ND-3 | LJF | 61 | F | M5 | 96.2 | 1.17 | 55 | 8 | 35 |
| ND-4 | ZZH | 22 | M | M4 | 36.16 | 1.49 | 54 | 21 | 93 |
| ND-5 | ZL | 40 | M | M5 | 45.23 | 2.01 | 77 | 61 | 80 |
| ND-6 | SSS | 38 | F | M3 | 2.15 | 4.31 | 142 | 128 | 79 |
| ND-7 | CXH | 28 | M | M5 | 14.59 | 3.19 | 96 | 21 | 81 |
| ND-8 | XYP | 53 | F | M3 | 17.46 | 2.79 | 89 | 10 | 90 |
| ND-9 | SY | 46 | M | M5 | 49.41 | 2.4 | 74 | 28 | 96 |
| ND-10 | GHY | 21 | F | M5 | 3.76 | 1.95 | 66 | 39 | 95 |
| ND-11 | LXJ | 53 | M | M4 | 65.87 | 1.14 | 57 | 110 | 63 |
| ND-12 | SQC | 52 | M | M3 | 31.14 | 2.34 | 73 | 51 | 85 |
| ND-13 | WJM | 34 | F | M3 | 190 | 2.74 | 102 | 54 | 95 |
| ND-14 | YYS | 51 | M | M5 | 9.56 | 1.25 | 46 | 30 | 78 |
| ND-15 | HYQ | 49 | F | M5 | 89.17 | 3.46 | 93 | 126 | 91 |
| ND-16 | LLM | 57 | M | M3 | 92.21 | 2.86 | 79 | 64 | 93 |
| ND-17 | ZYJ | 17 | F | M5 | 356 | 1.77 | 57 | 41 | 86 |
| ND-18 | MGQ | 25 | F | M3 | 12 | 3 | 80 | 60 | 94 |
| ND-19 | JK | 40 | M | M5 | 11 | 2 | 75 | 65 | 90 |
| ND-20 | CXF | 21 | F | AML | 14.09 | 4 | 66 | 77 | 80 |
| ND-21 | ZY | 43 | F | M1 | 45.59 | 2.63 | 89 | 48 | 42 |
| ND-22 | LWQ | 43 | M | M3 | 54.52 | 2.22 | 60 | 60 | 37 |
| ND-23 | LYG | 50 | F | M3 | 29.3 | 2.14 | 81 | 56 | 96 |
| ND-24 | LXJ | 41 | M | AML | 32.47 | 3.22 | 109 | 17 | 98 |
| ND-25 | ZYJ | 57 | F | M4 | 72.81 | 1.98 | 70 | 53 | 86 |
| ND-26 | LXL | 17 | F | M2 | 48.93 | 3.94 | 129 | 24 | 64 |
| ND-27 | YN | 29 | F | M3 | 6.09 | 4.4 | 135 | 191 | 34 |
| ND-28 | ZKG | 25 | M | M2 | 3.93 | 2.06 | 68 | 35 | 28 |
| ND-29 | LLH | 83 | M | AML | 6 | 3 | 67 | 78 | 78 |
| ND-30 | JWB | 43 | M | AML | 19.67 | 2.39 | 89 | 506 | 49 |
| ND-31 | KFY | 45 | M | M3 | 4.26 | 2.78 | 84 | 10 | 91 |
| ND-32 | WHR | 68 | F | M4 | 173.41 | 3.51 | 100 | 22 | 93 |
| ND-33 | ZL | 59 | F | AML | 1.5 | 2.2 | 65 | 72 | 43 |
| ND-34 | DW | 34 | F | M5 | 19.25 | 1.86 | 65 | 47 | 78 |
| ND-35 | QYH | 33 | M | M3 | 73.06 | 2.55 | 90 | 37 | 96 |
| ND-36 | ZHM | 57 | F | M4 | 223.4 | 2.77 | 82 | 35 | 82 |
| ND-37 | WF | 43 | F | M5 | 16.26 | 1.35 | 55 | 6 | 90 |
| ND-38 | LYX | 29 | F | M5 | 46.51 | 1.03 | 57 | 35 | 91 |
| ND-39 | LKL | 44 | F | M5 | 1.21 | 2.31 | 75 | 106 | 62 |
| ND-40 | LXY | 65 | F | M5 | 16.13 | 2.05 | 65 | 105 | 81 |
| ND-41 | LWJ | 46 | M | M3 | 104.89 | 2 | 66 | 78 | 93 |
| ND-42 | WYL | 13 | F | M3 | 1.63 | 2.34 | 75 | 33 | 92 |
| ND-43 | LY | 55 | M | M1 | 2.73 | 2.37 | 85 | 8 | 92 |
| ND-44 | WXY | 23 | F | M5 | 2.2 | 1.83 | 65 | 35 | 90 |
| ND-45 | XGX | 18 | F | M4 | 14.57 | 2.18 | 70 | 187 | 49 |
| ND-46 | LHL | 46 | F | M5 | 15.05 | 3.38 | 115 | 43 | 85 |
| ND-47 | JYX | 39 | M | M3 | 5.38 | 3.49 | 92 | 10 | 92 |
| ND-48 | ZY | 42 | F | M3 | 0.8 | 1.99 | 71 | 23 | 80 |
| ND-49 | PZQ | 43 | M | AML | 54.12 | 2.22 | 66 | 76 | 37 |
| ND-50 | WCJ | 44 | M | M5 | 1.21 | 2.01 | 80 | 90 | 62 |
| ND-51 | LJT | 65 | M | M5 | 17.13 | 2.05 | 65 | 115 | 80 |
| ND-52 | WXH | 45 | F | M5 | 7.57 | 2.23 | 87 | 44 | 90 |
| ND-53 | CL | 46 | F | M4 | 39.58 | 3.45 | 86 | 24 | 90 |
| ND-54 | GFS | 28 | M | M3 | 2.78 | 2.09 | 68 | 44 | 92 |
| ND-56 | RW | 37 | F | M5 | 32.28 | 1.61 | 59 | 59 | 95 |
| ND-57 | YCL | 32 | F | M3 | 5.3 | 1.42 | 65 | 8 | 88 |
| ND-58 | JZZ | 77 | F | M5 | 2.71 | 1.77 | 68 | 12 | 84 |
| ND-59 | FZS | 75 | M | AML | 27.13 | 2.05 | 65 | 85 | 60 |
| ND-60 | SXJ | 18 | F | AML(MDS) | 17.57 | 2.23 | 87 | 44 | 90 |
| ND-61 | SRY | 56 | F | AML | 9.58 | 3.45 | 86 | 24 | 90 |
| ND-62 | WCS | 45 | M | M5 | 12.78 | 2.09 | 68 | 44 | 92 |
| ND-63 | ZJG | 55 | M | M5 | 22.28 | 1.61 | 59 | 59 | 95 |
| ND-64 | ZLQ | 65 | F | M5 | 15.3 | 1.42 | 65 | 8 | 88 |
| ND-65 | YSF | 72 | F | M4 | 22.71 | 1.77 | 68 | 12 | 84 |
| ND-66 | ZYD | 56 | M | AML(EF) | 7.13 | 2.05 | 65 | 85 | 45 |
| ND-67 | NKY | 61 | F | AML(MPN) | 18.57 | 2.23 | 87 | 44 | 90 |
| ND-68 | WJL | 49 | M | AML(EF) | 1.12 | 4.1 | 90 | 67 | 43 |

Remarks: ND, newly diagnosed; CR, complete remission; WBC, white blood cells; RBC, red blood cells; HGB, haemoglobin; PLT, blood platelet; BM, bone marrow.


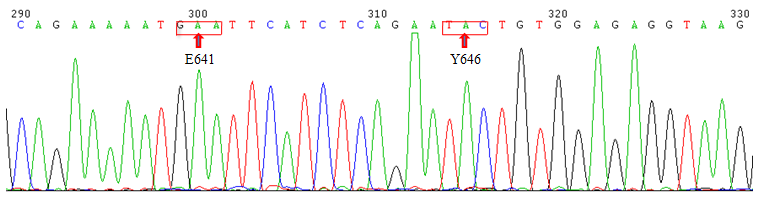


**Supplementary Figure 1**. The representative Sanger sequencing result of E641 and Y646 mutation hotspots on exon 16 in the EZH2 gene in ND AML patients.
